# Nutrients Turned into Toxins: Microbiota Modulation of Nutrient Properties in Chronic Kidney Disease

**DOI:** 10.3390/nu9050489

**Published:** 2017-05-12

**Authors:** Raul Fernandez-Prado, Raquel Esteras, Maria Vanessa Perez-Gomez, Carolina Gracia-Iguacel, Emilio Gonzalez-Parra, Ana B. Sanz, Alberto Ortiz, Maria Dolores Sanchez-Niño

**Affiliations:** Raul Fernandez-Prado, IIS-Fundación Jiménez Díaz-Universidad Autónoma de Madrid, 28040 Madrid, Spain; raul.fernandezp@quironsalud.es (R.F.-P.); raquel.esteras@quironsalud.es (R.E.); MVANESSA@fjd.es (M.V.P.-G.); CGraciaI@quironsalud.es (C.G.-I.); egparra@quironsalud.es (E.G.-P.); asanz@fjd.es (A.B.S.); aortiz@fjd.es (A.O.)

**Keywords:** chronic kidney disease, caramboxin, microbiota, choline, carnitine, tryptophan, tyrosine, trimethylamine N-Oxide (TMAO), p-cresyl sulfate, indoxyl sulfate, gut–kidney axis

## Abstract

In chronic kidney disease (CKD), accumulation of uremic toxins is associated with an increased risk of death. Some uremic toxins are ingested with the diet, such as phosphate and star fruit-derived caramboxin. Others result from nutrient processing by gut microbiota, yielding precursors of uremic toxins or uremic toxins themselves. These nutrients include l-carnitine, choline/phosphatidylcholine, tryptophan and tyrosine, which are also sold over-the-counter as nutritional supplements. Physicians and patients alike should be aware that, in CKD patients, the use of these supplements may lead to potentially toxic effects. Unfortunately, most patients with CKD are not aware of their condition. Some of the dietary components may modify the gut microbiota, increasing the number of bacteria that process them to yield uremic toxins, such as trimethylamine N-Oxide (TMAO), p-cresyl sulfate, indoxyl sulfate and indole-3 acetic acid. Circulating levels of nutrient-derived uremic toxins are associated to increased risk of death and cardiovascular disease and there is evidence that this association may be causal. Future developments may include maneuvers to modify gut processing or absorption of these nutrients or derivatives to improve CKD patient outcomes.

## 1. CKD and Uremic Toxins

The prevalence of chronic kidney disease (CKD) hovers around 5–15% of the adult population and increases with age [1,2]. However, CKD frequently remains undiagnosed: it has been estimated that 9% of adults over the age of 80 years have been diagnosed with CKD, but around 60% have undiagnosed CKD [3]. For the past 20 years, CKD has been the second fastest growing cause of death worldwide, after HIV infection [4]. When the glomerular filtration rate (GFR) falls below 60 mL/min/1.73 m^2^, the risk of all-cause and cardiovascular death increases with decreasing GFR, peaking at 10- to 100-fold over the same-age general population in patients undergoing dialysis [5]. This is thought to result mainly from accumulation of uremic toxins [6]. For a solute to be considered a uremic toxin, it should meet two criteria, one related to the mechanism underlying accumulation in CKD and another related to the contribution to CKD manifestations. Thus, uremic toxins have been defined as solutes normally excreted by the kidneys that are retained in CKD and interact negatively with biologic functions [7,8]. The European Uremic Toxin (EUTOX) Working Group database presents encyclopedic data on 130 uremic toxins [9,10]. Interestingly, serum levels of some toxins are not well correlated with the levels of endogenous molecules excreted by glomerular filtration, such as creatinine. This may be explained by differences in the amount of ingested or generated toxins, on top of the generally shared reduction in clearance. In this regard, uremic retention solutes may represent molecules ingested with food, such as bisphenol A or caramboxin, may derive from nutrients, as discussed in this review, or from molecules generated during the normal functioning of the body, such as creatinine [9,11,12]. The current review focuses on uremic toxins that are primarily derived from ingested nutrients, either because the molecules are toxic in the biochemical form found in nutrients or because an intermediate metabolite is generated by the gut microbiota that is then processed to the uremic toxin. Thus, a lower ingestion of the nutrient precursors may favorably influence health in CKD patients. Since many CKD patients remain undiagnosed, a wider portion of the population and their doctors should be familiar with nutrients that may become toxins in the CKD context.

## 2. The Microbiota

**Figure 1:**
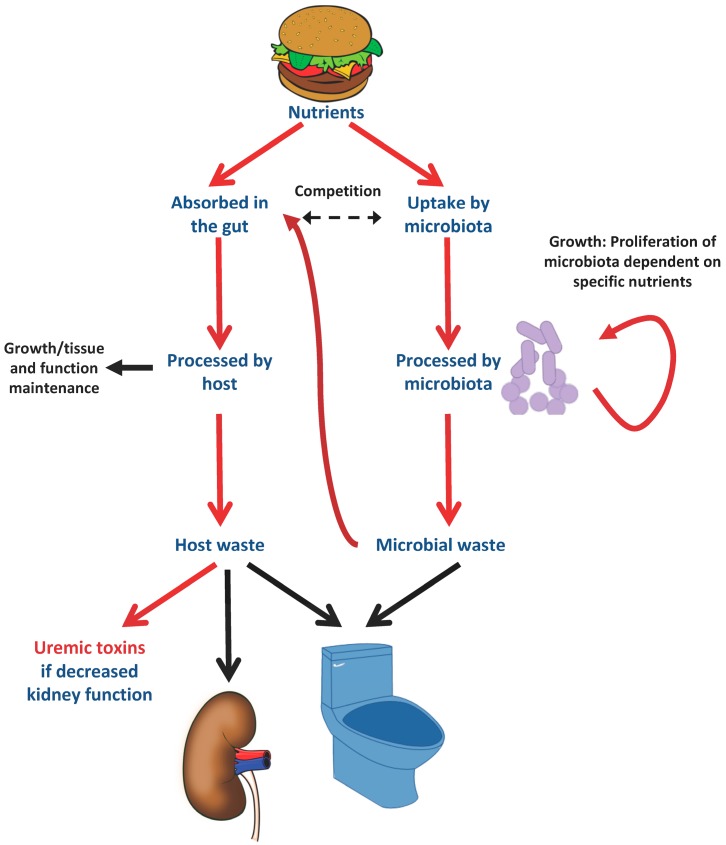
Microbiota–host interaction and modulation by dietary nutrients. When humans feed themselves, they are also feeding their microbiota. The precise nutrients ingested will favor the growth of bacteria that feed on ingested nutrients, in detriment of bacteria that feed on nutrients that a person is not ingesting. The microbiota competes with the host for certain nutrients, such l-carnitine. Thus, oral l-carnitine supplementation promotes the growth of l-carnitine metabolizing bacteria and may result in decreased l-carnitine absorption. Some waste molecules from bacterial feeding will be absorbed systemically and metabolized to uremic toxins that accumulate if not excreted in urine.

The microbiome was originally defined as “the ecological community of commensal, symbiotic, and pathogenic microorganisms that literally share our body space” [13]. However, the term microbiota usually refers to the microorganisms, while microbiome is generally used to refer to their genes. The human body contains around 10 times more bacteria than individual human cells, mostly in the gastrointestinal tract [6]. Essentially, when humans feed themselves, they are also feeding their microbiota. The precise nutrients ingested will favor the growth of bacteria that feed on ingested nutrients, in detriment of bacteria that feed on nutrients that a person is not ingesting (Figure 1). Bacteria may even compete with the host for nutrients as discussed below for l-carnitine. There is a bidirectional interaction between the kidney and the gut microbiota (the gut–kidney axis): bacteria may generate potentially toxic metabolites while CKD may impact the composition of the microbiota and damage the intestinal epithelial barrier, facilitating translocation of endotoxins and live bacteria from gut lumen into the bloodstream [14]. Bacteria are also exposed to molecules released from gut cells. In this regard, genetic defects in the gut cell inflammasome (e.g., nlrp6 deficiency) alter the gut microbiota [15]. Conversely, bacteria release metabolites that may be absorbed and provide benefit (e.g., vitamin K) or harm (e.g., uremic toxin precursors). The influence of such bacterially secreted molecules is larger than previously suspected. Thus, higher plasma levels or uremic toxins of gut origin were associated with evidence of more severe systemic inflammation in CKD patients [16]. The gut microbiota impacts host metabolism and is thought to play a key role in obesity, insulin resistance and cancer, among others [17]. Microbiome-associated human conditions are being studied by the Integrative Human Microbiome Project [18,19]. Some general patterns of gut bacteria associated with disease are emerging. Thus, a decrease in Bacteroidetes species and an increase in Firmicutes species has been associated with the obese state, and an adverse metabolic profile [20].

The microbiota may be altered by many factors and, thus, may contribute more or less to the accumulation of uremic toxins. These factors include the genetic background of the host; the amount of dietary fiber, proteins and toxin precursors; medical conditions including CKD and certain therapies, especially antibiotics [21]. In this regard, in CKD patients, Brachybacterium, Catenibacterium, Enterobacteriaceae, Halomonadaceae, Moraxellaceae, Nesterenkonia, Polyangiaceae, Pseudomonadaceae, and Thiothrix families were markedly increased, while in CKD rats, Lactobacillaceae and Prevotellaceae families were decreased [22]. From a enzymatic point of view, ESRD patients exhibited significant expansion of bacterial families possessing urease, uricase, and indole and p-cresol forming enzymes, and contraction of families possessing enzymes converting dietary fiber to short-chain fatty acids such as butyrate [23].

## 3. Food-Derived Uremic Toxins

Molecules found in food may be directly toxic. Some nutrients, such as phosphate or oxalate, may become toxic when accumulated in CKD. For other food components, such as caramboxin, there is no known nutritional benefit.

### 3.1. Phosphate

Around 1200 mg phosphate are ingested daily with food, of which around 900 mg are absorbed and must be excreted in urine [24]. Kidneys adjust serum phosphate levels and prevent phosphate accumulation. In CKD, phosphate accumulates and serum phosphate increases with decreasing GFR. Phosphate has been considered a uremic toxin associated with high cardiovascular risk in individuals with normal renal function and in CKD patients [24]. Serum phosphate in the high-normal range (normal range 2.5–4.5 mg/dL) is associated to a higher risk of cardiovascular disease and mortality [25,26,27,28,29], as well as to faster CKD progression [30]. In observational studies, the use of phosphate binders was associated with lower mortality in patients on hemodialysis [31].

Dietary phosphate content used to be closely related to protein content and phosphate associated to animal proteins is better absorbed than phosphate from vegetable sources. However, in Western societies, inorganic phosphate from soda and food additives represents a rich source of very readily absorbed phosphate, not always associated to protein content, and not always well labeled in the processed food nutritional information.

The association between increased serum phosphate or phosphate overload and cardiovascular risk could result from direct promotion of cardiovascular injury, including vascular calcification [32,33] or from adaptive mechanisms to excess phosphate. A positive phosphate balance activates the phosphatonins parathyroid hormone (PTH) and fibroblast growth factor-23 (FGF-23) that promote phosphaturia [34]. FGF-23 needs a cofactor, α-Klotho, to activate the FGFR1 receptor [35]. In CKD, kidney inflammation downregulates Klotho, resulting in FGF-23 resistance and the need or even higher FGF-23 levels [36,37]. Lack of functional FGF-23 or Klotho is associated with premature aging, atherosclerosis and vascular calcification, which is prevented by a low phosphate diet [38]. In addition, FGF-23 downregulates calcitriol synthesis by inhibiting proximal tubular 1α-hydroxylase [39] and increasing the 24-OH-hydroxylase activity that degrades calcitriol. Excess circulating FGF-23 and PTH, or reduced calcitriol levels, may also directly promote cardiovascular injury and interfere with antibacterial defenses [40,41,42,43,44].

### 3.2. Caramboxin

**Figure 2:**
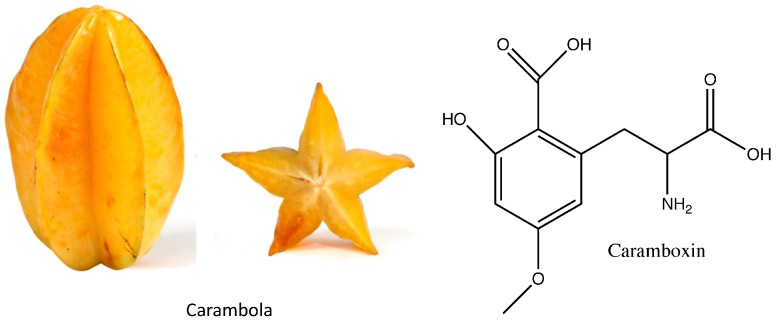
Carambola (star fruit) and caramboxin. CKD patients should be instructed to recognize and avoid carambola because of the risks it entails.

Caramboxin is a neurotoxin found in carambola (star fruit, *Averrhoa carambola*), a tropical fruit. Carambola has antioxidant properties and its use has been recommended to improve the lipid profile [45], depress pro-inflammation cytokines [46] and as a potential treatment for diabetes [47]. Carambola (Figure 2) may be found in supermarkets around the globe, but its toxicity in CKD patients remains poorly recognized by the general population and even less by those unaware of being CKD patients. The toxic effect of carambola was first reported in 1980 [48], but caramboxin was not described until 2013 [49]. Caramboxin is a nonpeptide amino acid, phenylalanine-like molecule that inhibits the GABAergic system through a glutamatergic ionotropic molecular action, having potent excitatory properties [49]. From 2000 to 2014 neurotoxicity related to carambola ingestion was reported in 110 patients, of whom 27 died [50]. The vast majority had CKD and developed neurotoxicity resulting from accumulation of caramboxin due to reduced renal excretion. However, the carambola fruit is also nephrotoxic and may cause both acute kidney injury (AKI) and neurotoxicity [51,52]. The amount of fruit consumed ranged from a glass of juice to 4 carambolas and symptoms started between 2 h and 2 days after ingestion. The initial presentations included uncontrollable hiccups (a typical manifestation found in over 90% of patients), vomiting, mental confusion, seizures, coma and death [50]. Daily hemodialysis or continuous renal replacement methods is the therapy of choice for severe cases [53]. While oxalate nephropathy has been observed in renal biopsies from patients with carambola nephrotoxicity, the potential contribution of caramboxin to kidney injury has not been studied. In this regard, carambola juice is directly toxic for cultured tubular cells [54] and glutamatergic signaling was identified as a potential contributor to AKI by a systems biology, kidney tissue proteomics-based approach [55].

### 3.3. Oxalate

Plasma oxalate is increased in CKD patients and renal replacement therapy is not effective for permanently reducing oxalate levels [56]. As kidney function declines, the colon also gains importance in the homeostasis and disposal of oxalate. Oxalate colonic secretion may be increased by drugs increasing the expression of cAMP and by probiotics (e.g., *Oxalobacter formigenes*) [57]. Excess oxalate may become deposited and become nephrotoxic itself [58]. Indeed, as noted above, oxalate is thought to be the nephrotoxic component in carambola.

## 4. Nutrients as Uremic Toxins Precursors via the Microbiota

**Figure 3:**
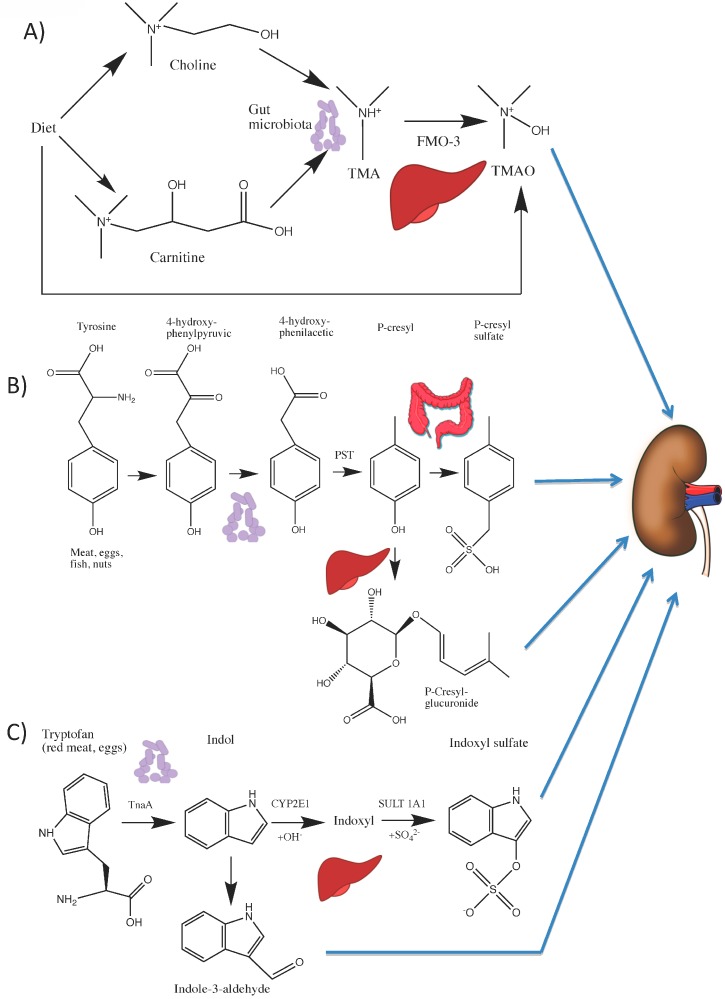
From nutrients to toxins: (**A**) Metabolic pathways for generation of the uremic toxin TMAO from dietary l-carnitine and choline; (**B**) metabolic pathways for generation of the uremic toxins p-cresyl-sulfate and p-cresyl-glucuronide from dietary tyrosine; and (**C**) metabolic pathways for generation of the uremic toxins indoxyl sulfate and indole-3-aldehyde from dietary tryptophan. The uremic toxins thus generated are excreted by the kidneys in healthy subjects but accumulate as uremic toxins in individuals with CKD.

**Figure 4:**
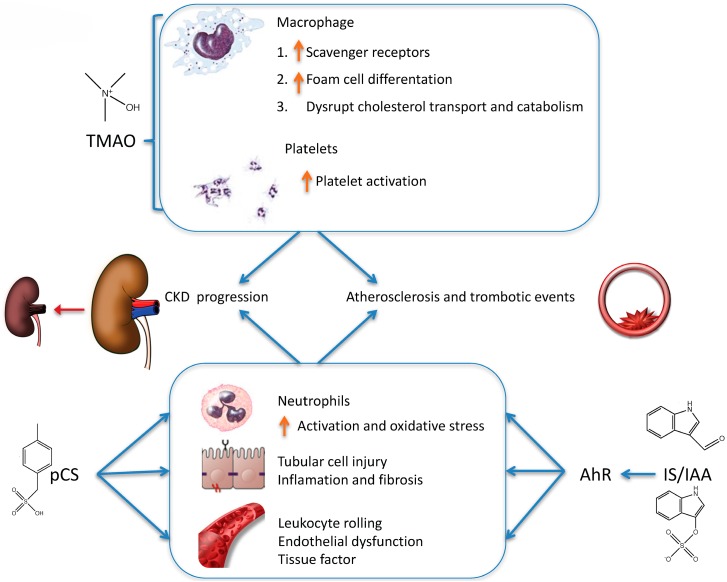
Mechanisms of toxicity for nutrient-derived uremic toxins. The molecular mechanisms of uremic toxin toxicity are starting to be elucidated. The figure represents some key recently described pathways by which they may contribute to the two key consequences of CKD: CKD progression and accelerated cardiovascular aging.

Some nutrients from food are processed by the gut microbiota to generate uremic toxins or precursors that are metabolized to toxins in the body. Trimethylamine N-Oxide (TMAO), p-cresyl-sulfate (pCS), indoxyl-sulfate (IS) and indole-3 acetic acid (IAA) are key uremic toxins originating from dietary nutrients (Figure 3 and Figure 4).

### 4.1. Choline and l-carnitine Are Metabolized to TMAO

**Choline** is an essential nutrient and quaternary ammonium salt containing the *N*,*N*,*N*-trimethylethanolammonium cation. Choline is phosphorylated by choline kinase, generating phosphatidylcholine that incorporates into cell membranes. In the liver and kidney, choline is oxidized to betaine, which serves as a methyl donor. In neurons, choline acetyltransferase catalyzes its incorporation into the neurotransmitter acetylcholine [59]. There is an endogenous pathway for the de novo biosynthesis of choline via the sequential methylation of phosphatidylethanolamine using *S*-adenosylmethionine as the methyl donor. Thus, the demand for dietary choline is modified by metabolic methyl-exchange relationships between choline and three nutrients: methionine, folate, and vitamin B12 [60]. In this regard, choline is considered an essential nutrient by the Food and Nutrition Board of the Institute of Medicine, which recommends an adequate intake of 550 mg/day for adult men and 425 mg/day for adult women [61], although ingestion of around 1 g/day choline is common in healthy individuals. Lecithins added during food processing may increase the average daily intake of phosphatidylcholine [61]. Choline is widely distributed in foods, mostly as phosphatidylcholine in cell membranes and inadequate intake is unusual except in strict vegetarians who consume no milk or eggs. Foods especially rich in choline compounds include egg yolk (800 mg/100 g), kidney and liver (400 mg/100 g), chocolate- and protein-based beverages (300 mg/100 g), salmon and soy protein (200 mg/g), powdered milk (170 mg/100 g) and meat (150 mg/g) [62]. Choline is freely filtered at the glomerulus, but most (97%) filtered choline is reabsorbed by proximal tubules, resulting in a renal clearance of around 2 mL/min. Higher choline levels are found in CKD patients, especially in dialysis patients [63]. Choline is cleared by dialysis and plasma free choline concentration falls during hemodialysis but returns to baseline levels 6 h later [63].

Choline is available as a dietary supplement as choline chloride or choline bitartrate and as lecithin, which usually contains approximately 25% phosphatidylcholine or 3–4% choline by weight. Choline supplements are marketed for liver health, memory and improved physical performance. In this regard, choline supplementation has been considered for a variety of conditions, including exercising, pregnancy and Alzheimer’s disease [64,65]. Plasma choline concentration are reduced approximately 40% during marathon running [66]. However, preventing the decrease in plasma choline by short-term lecithin supplementation prior to a marathon failed to improve performance [67]. Furthermore, a recent systematic review concluded that evidence to confirm the suggested effects of choline on health in different stages of life is scarce [68]. Pharmacological doses of choline chloride (10 g/day) were associated to nausea, diarrhea, and a small fall in blood pressure in patients studied in the 70 s [69].

**l-carnitine** is an amino acid derivative that transports cytosolic long-chain fatty acids as acylcarnitines across the inner mitochondrial membrane for β-oxidation to generate ATP [70]. l-carnitine is obtained from dietary sources and from endogenous biosynthesis from lysine and methionine in kidneys and liver [71,72]. Meat has the highest contents of l-carnitine, including kangaroo meat (637 mg/100 g of dry weight), horse meat (423 mg/100 g) and beef (139 mg/100 g). The amount of l-carnitine in milk products ranges from 1 to 43 mg/100 g of dry matter. Vegetables and fruits contain <5 mg l-carnitine/100 g of dry matter. Mushrooms are richer in l-carnitine than plants. The amount of l-carnitine (53 mg/100 g dry matter) in *Pleureotus ostreatus* equals approximately 100 g of minced pork [73]. Absorption of orally administered l-carnitine is very variable and ranges from 5% to 18% for pharmacological doses to up to 75% for dietary l-carnitine. The low bioavailability depends both on transport kinetics and on l-carnitine metabolism by intestinal bacteria as the number of l-carnitine processing bacteria increases when l-carnitine is abundant [74,75]. As it is the case for choline, the renal clearance of l-carnitine (1–3 mL/min) is low because of extensive tubular reabsorption. However, since there is a threshold concentration for tubular reabsorption, renal clearance is much higher following high dose intravenous l-carnitine [76].

Carnitine is an essential nutrient only in infants, according to the Food and Nutrition Board of the Institute of Medicine. l-Carnitine supplements are indicated to treat primary systemic carnitine deficiency, an ultrarare disease resulting from genetic defects in the high-affinity plasma membrane carnitine-carrier_in_ leading to renal carnitine wasting [77]. However, athletes may take l-carnitine supplements to boost performance and these supplements are marketed online to “burn fat”.

More than 99% of the carnitine pool is located outside of plasma and, thus, plasma concentration is not a good marker of carnitine availability. During hemodialysis, large amounts of l-carnitine are lost in dialysate. Chronic hemodialysis may lead to progressive l-carnitine deficiency through a combination of loss of l-carnitine in dialysate and decreased l-carnitine synthesis by the injured kidney [78]. However, low renal clearance of short-chain acylcarnitines results in a high acylcarnitine:l-carnitine ratio in CKD patients. Intravenous and oral l-carnitine for chronic renal failure anemia and intradialytic hypotension are licensed and reimbursed [79], although routine l-carnitine supplementation for hemodialysis patients is not recommended by clinical guidelines, based on lack of definitive evidence of benefit [80,81,82,83,84]. Despite this lack of endorsement of the practice, physicians may prescribe l-carnitine supplementation to hemodialysis patients given the potential benefit and perceived lack of adverse effects [85]. However, as discussed below, microbiota processing of oral l-carnitine in the gut may generate uremic toxins. In this regard, a meta-analysis suggested that oral l-carnitine increased serum cholesterol, but intravenous l-carnitine did not [86]. Different biological effects dependent on gut microbiota generation of toxins between oral and intravenous l-carnitine supplementation may have contributed to contradictory evidence [70].

**TMAO** is an amine oxide from dietary seafood. However, TMAO is mainly generated from dietary choline and d,l-carnitine. Choline and d,l-Carnitine are metabolized by gut microbiota to trimethylamine (TMA) [87], which is absorbed and oxidized by hepatic flavin monooxygenase-3 (FMO) to TMAO, which is eventually excreted, mainly in urine, but also in sweat and exhaled air (Figure 3A) [88,89,90]. Genetic deficiencies in FMO prevent conversion of maladorous TMA to odorless TMAO, resulting in trimethylaminuria or “fish odor syndrome” [91]. Plasma TMA, dimethylamine (DMA) and TMAO concentrations are elevated in uremia [92,93], because of decreased GFR with or without derangements in the gut microbiota [22]. Increased urinary TMAO levels in CKD patients may represent local renal TMAO synthesis or renal excretion of circulating TMAO [94], while hemodialysis efficiently removes TMAO from plasma [92].

Ingestion of precursors, genetic differences in the activity of enzymes involved in TMAO metabolism and the specific composition of the microbiota may account for large intra and interindividual differences in plasma TMAO levels among patients on dialysis [88,91,95,96]. Dose-dependent increases in circulating TMA and TMAO levels were observed in healthy volunteers following oral l-carnitine dosing [76]. In hemodialysis patients, oral l-carnitine did not change plasma TMA, but doubled plasma TMAO concentrations within two weeks [97]. By contrast, a report from Japan, a country where fish is an important dietary source of TMAO; observed an increase in circulating TMA levels but not of TMAO following oral l-carnitine [98]. Furthermore, predialysis TMAO levels in Japanese hemodialysis patients were in the range found in healthy volunteers. However, TMAO was increased in Australian patients on hemodialysis not supplemented with l-carnitine and in the absence of fish ingestion for 48 h [9,92]. These differences are interesting given the long survival of Japanese dialysis patients and their low incidence of cardiovascular diseases [99]. Regarding potential ethnic differences, TMAO concentration did not differ between white and black hemodialysis patients. However, TMAO was associated with an increased risk of cardiac or all-cause death in whites, but not in blacks [100]. A phosphatidylcholine challenge (ingestion of two hard-boiled eggs) also increased plasma TMAO [101]. This response was suppressed by the administration of antibiotics to modify the microbiota and reappeared after withdrawal of antibiotics. In mice, antibiotics also prevented the increase in TMAO in response to oral l-carnitine and increased circulating l-carnitine levels following oral supplementation, presumably because of lower bacterial catabolism into TMA [88]. In mice, specific bacterial taxa in the gut were associated with higher TMAO levels and were altered in response to chronic dietary l-carnitine supplementation, resulting in markedly enhanced synthesis of TMA and TMAO. Thus, dietary l-carnitine itself induced the microbiota capacity to metabolize l-carnitine into TMA. TMAO itself favors the growth of anaerobic intestinal bacteria as Enterobacteriaceae and inhibits the growth of others as *Staphylococcus aureus* [90]. In this regard, omnivorous humans (eating red meat) produced more TMAO upon oral l-carnitine challenge than did vegans or vegetarians. However, an oral 250 mg labeled l-carnitine challenge, had little impact on endogenous TMAO levels in subjects with normal renal function [88]. Given the large quantities of l-carnitine in red meats, l-carnitine and the microbiota may contribute to the causal link between high consumption of red meat and cardiovascular and renal risk [88]. Red meat intake, but not protein intake or protein from other sources, strongly associated with ESRD risk in a dose-dependent manner and substituting alternative sources of protein may reduce the incidence of ESRD [102].

Metabolomics identified plasma TMAO as a predictor of cardiovascular risk that was confirmed in an independent large clinical cohort [103]. High TMAO levels were associated with increased risk of mortality in CKD patients and with increased risk of renal disease progression in an animal model [93]. Higher TMAO levels are associated with a higher risk of major adverse cardiovascular events such as death, myocardial infarction or stroke [88,101,103,104,105,106], an advanced cardiometabolic risk profile [107], and higher incidence and poorer prognosis of heart failure [108,109]. Supplementation with l-carnitine or TMAO promoted atherosclerosis in mice [101]. The pro-atherogenic effect of TMAO may be related to upregulation of macrophage scavenger receptors involved in atherosclerosis, promoting foam cell differentiation [103] or to reduced reverse cholesterol transport and reduced expression of Cyp7a1, the rate limiting step in the catabolism of cholesterol [88]. TMAO also stabilizes the folded state of diverse proteins, functioning as a chemical chaperone [110] and enhances stimulus-dependent platelet activation from multiple agonists through increased Ca(2+) release from intracellular stores [111]. The contribution to any toxic effects of the chaperone function of TMAO is poorly characterized. TMAO stabilizes proteins through at least two mechanisms: (i) decreased hydrogen bonding ability of water and hence the stability of the unfolded state; and (ii) acting as a molecular crowder, that can increase the stability of the folded state via the excluded volume effect [110]. While initial studies focused on potential benefits derived from this chemical chaperone activity [112,113], more recent publications draw attention to a potential pathological impact in the absence of stressors, as potentiating the aggregation of amyloidogenic intrinsically disordered peptides, such as Aβ42 [114]. By contrast, the potential pathological contribution of increased platelet aggregation is supported by epidemiological studies of an association between plasma TMAO and incident (three years) thrombosis (heart attack and stroke) risk and by animal studies employing dietary choline or TMAO, germ-free mice, and microbial transplantation that confirmed a role for gut microbiota and TMAO in modulating platelet hyperresponsiveness and thrombosis potential [111].

### 4.2. Tryptophan Is Metabolized to Indoxyl Sulfate (IS) and Indole-3 Acetic Acid (IAA)

**Tryptophan** (Trp, W) is an essential amino acid in humans, meaning that it cannot be synthesized and must be obtained from the diet. The FAO estimated the daily requirements in 3.5 mg/kg/day in adults [115]. It is abundant in egg white (1.4 g/100 g of food), concentrated soy products and the cyanobacteria spirulina (*Arthrospira platensis*) (0.93 g/100 g), milk and cheese (0.5 g/100 g) and meat (0.4 g/100 g). Tryptophan is a precursor for niacin and melatonin, thus taking part in the regulation of sleep disorders. Additionally, it is metabolized to 5-hydroxytryptophan which is subsequently converted into the neurotransmitter serotonin, a regulator of depression. Gut bacteria expressing tryptophanase metabolize tryptophan to indole and derivatives.

The kidneys excrete tryptophan derivatives and metabolize tryptophan via the kynurenine pathway. Higher levels of tryptophan in ESRD patients are associated with lower total cholesterol and systolic blood pressure [116]. In hemodialysis patients, tryptophan catabolites of the kynurenine pathway are increased perhaps due to an enhanced activity of tryptophan-degrading enzyme indoleamine 2,3-dioxygenase (IDO) inducible by pro-inflammatory stimuli [117,118]. Parkinson’s disease, motor neuron disease and multiple sclerosis have been associated with the kynurenine pathway [119].

Tryptophan is sold over the counter as a dietary supplement for use as an antidepressant, anxiolytic and sleep aid. It has been proposed that consumption of tryptophan may improve depression, mood and anxiety disorders by increasing serotonin in the brain. However, this use of tryptophan is not supported by scientific evidence [120].

A variety of uremic toxins result from tryptophan metabolism, including indolic uremic toxins (IS, IAA, and indoxyl-β-d-glucuronide) and toxins from the kynurenine pathway (kynurenine, kynurenic acid, anthranilic acid, 3-hydroxykynurenine, 3-hydroxyanthranilic acid, and quinolinic acid) [121]. They are ligands of the transcription factor aryl hydrocarbon receptor (AhR), also known as the dioxin receptor. AhR activation is known to mediate cardiotoxicity, vascular inflammation, and a procoagulant and prooxidant phenotype of vascular cells [121].

**Indoxyl Sulfate** (**IS**) is an indole derivative that accumulates in uremia [122,123]. Bacterial tryptophanase (TnaA) from Citrobacter, Escherichia, and Proteus, among others, process tryptophan to indole [124]. Indole is absorbed and is oxidized in the liver to indoxyl by cytochrome p450-2E1 and then sulfated by sulfotransferase 1A1 to form IS [123] (Figure 3C). Lactobacillus species metabolize tryptophan into indole-3-aldehyde that can also be metabolized by the liver into IS [125,126].

The uremic toxicity of IS was recently reviewed and a role in vascular and renal disease progression was suggested [127]. In CKD patients, IS levels were inversely related with renal function and directly related to aortic calcification, and predicted all-cause and cardiovascular mortality [128], although not in all studies [129] and the association with CKD progression is weaker than for pCS [129]. IS may favor fibrosis and cellular senescence through activation of NADPH oxidase to generate reactive oxygen species (ROS), NFκB activation, Klotho downregulation, TGF-β1 secretion, and inflammatory molecule (ICAM-1 and MCP-1) and senescence-related molecule (p21) expression [123,130]. IS may also be nephrotoxic, as it induced ER stress in tubular cells and inhibited cell proliferation [131]. In mice, IS suppressed endothelial progenitor cell-mediated neovascularization in ischemic limbs [132]. IS activates the aryl hydrocarbon receptor (AHR) that in turn interacts directly with and stabilizes functional tissue factor, preventing its ubiquitination and degradation and favoring thrombosis [133,134,135]. IS also enhanced platelet activities, including responses to collagen and thrombin, platelet-derived microparticles and platelet-monocyte aggregates, favoring thrombosis [136] and is a risk factor for dialysis graft thrombosis after endovascular interventions [137]. By contrast, AHR antagonists decreased tissue factor and were anti-thrombotic in uremia [133]. In the rat peritoneal vascular bed, IS disrupted the glycocalyx and induced strong leukocyte adhesion, enhanced extravasation, and interrupted blood flow [138]. IS also interferes with erythropoietin production, potentially favoring anemia in CKD patients [139], increases oxidative stress in osteoblasts, inducing PTH resistance and favoring adynamic bone disease [140] and promoted insulin resistance [141]. IS accumulated in muscles in murine CKD and caused mitochondrial dysfunction and decreased ATP availability in muscle cells. In CKD patients plasma IS inversely associated with and skeletal muscle mass [142].

**Indole-3 Acetic Acid** (**IAA**) predicted mortality and cardiovascular events in CKD patients. In cultured human endothelial cells, IAA activated an inflammatory AhR/p38MAPK/NF-κB pathway that induced the proinflammatory enzyme cyclooxygenase-2, and increased ROS production and tissue factor expression [135,143].

### 4.3. Tyrosine Is Metabolized to p-Cresyl Sulfate (pCS)

**l-tyrosine** (para-tyrosine, Tyr, Y) or 4-hydroxyphenylalanine is considered a conditionally indispensable amino acid that can be synthesized from the essential amino acid l-phenylalanine in the liver. l-tyrosine is a precursor of several biologically active molecules, including catecholamine neurotransmitters, hormones, and melanin [144,145]. High amounts of tyrosine are found in egg white and dried soy products (3 g/100 g), spirulina (2.6 g/100 g), milk and cheese (1.5–2.5 g/100 g) and meat (1.3 g/100 g) [62]. The FAO estimated the daily requirements of tyrosine and phenylalanine at 14 mg/kg/day in adults, around 1 g/day [115]. However, in the 1988–1994 NHANES III, the mean daily intake of tyrosine was 2.8 g/day. Young men had the highest intake at 6.4 g/day. This suggests that tyrosine supplementation is usually unnecessary. Indeed, tyrosine deficiency is rare. In hemodialysis patients, tyrosine levels were lower than in healthy controls and decreased with decreasing GFR [146,147].

A recent Cochrane Database review concluded that no recommendations can be made on tyrosine supplementation for routine clinical practice [148]. However, a number of studies have employed pharmacological doses of tyrosine, in the 150–500 mg/kg/day range [149]. In this regard, tyrosine is available as a dietary supplement. Reports on the effectiveness of tyrosine supplementation vary considerably, with some studies finding beneficial effects, whereas others do not. A recent review concluded that the potential of tyrosine supplementation for enhancing physical exercise capacity seems minimal. In contrast, tyrosine seemed to effectively enhance cognitive performance, particularly in short-term stressful and/or cognitively demanding situations, but only when neurotransmitter function is intact and dopamine and/or norepinephrine is temporarily depleted [149]. Studies investigating the role of tyrosine in cognitive function were performed in young adult populations and mostly under special conditions. Research in elderly populations is warranted [150]. Meta- and ortho-tyrosine are structural isomers of l-tyrosine (para-tyrosine) generated under conditions of oxidative stress and l-tyrosine supplementation has been suggested to potentially protect from oxidative stress-related diseases by competing with meta- and ortho-tyrosine [151].

Breakdown of tyrosine and phenylalanine by anaerobic intestinal bacteria, such as Bacteroides, Bifidobacterium, Lactobacillus, Enterobacter and Clostridium generate phenols as phenyl acetic acid and p-cresol [124]. p-cresol is absorbed in the gut and metabolized to **pCS** in enterocytes or to p-cresyl-glucuronide (**pCG**) in the liver [152,153] (Figure 3B). Although the older literature refers to increased p-cresol concentrations in CKD patients, this is an artifact generated by breakdown of pCS to p-cresol during processing for the assay. In fact, free p-cresol concentrations are undetectable or very low in plasma [152], while total serum pCS concentrations are four-fold higher than those of pCG [154]. While pCS is mostly protein-bound, only 8% of pCG is protein-bound, allowing a better clearance by dialysis. Moreover, pCG is less toxic than pCS on leucocytes and tubular cells [154,155]. In rat peritoneal vascular beds, pCS increased leukocyte rolling, while the combination of pCS and pCG impaired blood flow and vascular leakage but did not further enhance leukocyte rolling [156]. pCS has pro-apoptotic and pro-inflammatory effects on tubular cells [155]. Furthermore, in cultured tubular cells, pCS increased TGF-β1 secretion and induced some features of EMT [130] and serum pCS levels were predictive of CKD progression and mortality [129,157]. The uremic toxicity of pCS was recently reviewed and a role in vascular and renal disease progression was suggested [127].

## 5. Potential Therapeutic Implications

The main message from this review is that physicians considering supplementing their patients with nutrients that may potentially yield uremic toxins should first assess the renal function of their patients. Additionally, physicians should also explore the dietary habits of their CKD patients, specifically asking about intake of nutritional supplements. On top of this, there is very active research on maneuvers to modify gut processing or absorption of these nutrients or derivatives to improve CKD patient outcome. AST-120 was approved for clinical use in Japanese CKD patients in 1991 to retard CKD progression. It adsorbs indole in the gut and lowers IS levels. However, it failed to prevent CKD progression in a large multinational trial [158,159]. In addition to the decrease in uremic toxins discussed above, depletion of the microbiota with broad-spectrum antibiotics decreased inflammation and attenuated renal damage caused by renal ischemia-reperfusion in mice while fecal transplants in antibiotic-treated mice abolished the protective effect of antibiotics [160]. However, antibiotics cannot be promoted to lower uremic toxin levels since the expected risks far exceed the foreseeable benefits.

Modulating the colon microbiota of CKD patients has been suggested as a means to regulate the synthesis of key uremic toxins. There are different ways to modulate the gut microbiota. A key one is a change in dietary habits, as suggested by l-carnitine and TMAO studies discussed above. The ClC-2 chloride channel activator lubiprostone, a therapy for constipation, favored the recovery of the levels of the Lactobacillaceae family and Prevotella genus, which were significantly reduced in renal failure mice, and decreased plasma IS, although it is unclear whether this was related to changes in the microbiota or to better preserved kidney function, as mice were protected from kidney injury [161]. Prebiotics, probiotics and synbiotics have been tested, aiming at improving the composition or activity of intestinal microbiota aimed at increasing saccharolytic bacteria and reducing proteolytic bacteria. However, there are no quality intervention studies demonstrating clinical benefit [6,162,163,164]. There are only small studies assessing the effect on uremic toxin levels. Prebiotics are non-digestible food ingredients that may change the composition or activity of intestinal microbiota. Oligofructose inulin reduced p-cresol in hemodialysis patients but it did not modify IS [165]. Probiotics are “live-microorganisms”. Synbiotics are a combination of prebiotics and probiotics. In renal patients, synbiotics led to a reduction in pCS levels but not in IS levels and modified the stool microbiome, increasing bifidobacteria, in a proof-of-concept trial [166]. These results are consistent with another even smaller study in which a synbiotic combination of *Lactobacillus casei*, *Bifidobacterium breve* and galacto-oligosaccharide also decreased total p-cresol in hemodialysis patients [167]. An improved understanding of the gut microbial composition may allow the exploration of new potential treatment targets such as enzyme pathways [168].
